# Impact of modeling resin application techniques on the color stability and fluoride release from Giomer restorative material (an in vitro study)

**DOI:** 10.1186/s12903-026-08771-z

**Published:** 2026-06-05

**Authors:** Abdallah Maher Gaber Keshk, Sarah Diaa Shaheen, Asmaa Aly Mohamed Yassen

**Affiliations:** 1 Operative Dentistry Division, Conservative Surgery Department, College of Oral and Dental Surgery, Misr University for Science and Technology, 6th of October City, Egypt; 2 Operative Dentistry Department, Faculty of Dentistry, Suez University, Suez, Egypt; 3 Conservative Dentistry Department, Faculty of Dentistry, Cairo University, Cairo, Egypt

**Keywords:** Modeling resin, Giomer, Color change, Fluoride release

## Abstract

**Objectives:**

This in vitro study evaluated the effects of resin application methods on the color stability and fluoride release of a Giomer-based restorative material.

**Materials and methods:**

Seventy-two Beautifil LS Giomer specimens were divided into three Groups (*n* = 24; 12 for each test): surface modeling resin, interlayer modeling resin, and no resin (control). Baseline color and color change (ΔE00) after 14 days of coffee immersion (15 minutes, twice daily) were measured with a spectrophotometer. Fluoride release was measured by ion chromatography and recorded over 28 days. One-way ANOVA with Tukey’s post hoc test and paired t-tests were used for statistical analysis, with significance at *p* < 0.05.

**Results:**

At baseline, modeling resin application significantly influenced Giomer color parameters, with lower lightness values observed in the modeling resin Groups compared with the control (*p* < 0.05). After aging, surface modeling resin application showed significantly lower color changes and ΔE00 values than the control, with no significant difference between the two modeling resin techniques. Fluoride release over 28 days was comparable among Groups (*p* > 0.05), and no significant correlation was found between fluoride release and color change.

**Conclusion:**

Modeling resin affects baseline Giomer color and enhances color stability without impairing fluoride release, with no correlation between optical changes and fluoride release.

**Clinical relevance:**

Modeling resin application alters the baseline color characteristics of Giomer restorations, highlighting the importance of accurate shade selection. Surface application significantly improves color stability and resistance to staining without compromising fluoride release.

## Introduction

Glass ionomer cement (GIC) was introduced as a translucent, biocompatible restorative material. Its high fluoride release and chemical affinity to tooth structure make it useful as a restoration, luting cement, or base [1]. Conventional GICs have drawbacks, including dehydration, moisture sensitivity, a slow setting, and a rough surface texture. These factors may weaken the material, leading to clinical failures [2]. New materials address these issues, including resin-modified glass ionomer cements, Giomer, and high-viscosity glass ionomer cements [3–5].

Giomer is a hybrid esthetic material that represents a technological advancement in restorative dentistry. Compositionally, it is a resin-based restorative material containing Surface Pre-Reacted Glass-ionomer (S-PRG) fillers. Its benefits include fluoride release and recharge, acid resistance, anti-plaque action, dentin remineralization, and acid buffering ability. These features make it a promising esthetic restorative material, which is expected to provide long-term results [6].

Water sorption, dietary staining, and interactions with dental plaque can affect the color stability of any restorative material. Applying a modeling resin layer may alter the interaction of the restorative material with staining agents and oral environmental factors. Understanding these effects is essential for optimizing clinical performance, particularly in anterior restorations where color stability and esthetic durability are paramount [7–9].

Modeling resin was introduced to improve the restoration quality and reduce instrument stickiness. Many studies examined its mechanical and esthetic effects on the resin composite restorations [10, 11]. However, its impact on Giomer, including fluoride release, and its correlation with color stability, remain unassessed, and represent a true knowledge gap in the current dental literature.

This study aimed to investigate the cumulative fluoride release from Giomer and its color stability after modeling resin application. The study also evaluated the effect of application technique by comparing the modeling resin between layers or as a surface layer only.

This study began with the assumption that using a modeling resin would not impact Giomer’s color stability or its fluoride release. Additionally, it was assumed that applying modeling resin to all layers or only the last layer would have no effect.

## Materials and methods

The Giomer used was Beautiful II L.S (Shofu Inc., Kyoto, Japan), Shade A2. The wetting agent for all Groups was Ceramage modeling resin (Shofu Inc., Kyoto, Japan). Table 1 details the commercial name, specification, composition, lot number, and manufacturer of the tested resin-based materials.

**Table 1:** Materials used in this study, with their commercial name, specification, composition, batch number, and manufacturer

Commercial material	Specification	Composition	Lot number	Manufacturer
Ceramage	Modeling resin	Bis-GMA, UDMA. Dimethacrylate compounds: Monomers, Inorganic Fillers: Silica or alumina, Glass fillers (barium or strontium-based), Color pigments, Polymerization initiators, Stabilizers and antioxidants light-curing micro-hybrid composite with a ceramic portion of more than 73% micro-fine filler.	042399	Shofu Inc, Kyoto, Japan
Beautifil II LS	Giomer	Light-cured resin matrix (Bis-GMA, Bis-MPEPP, TEGDMA, UDMA), pigments, phoinitiator with surface pre-reacted glass fillers (S-PRG) based on fluoro-boro-alumino-silicate glass; filler content ≈ 82.9 wt% (≈ 68.6 vol%) with a particle size of 0.01- 4 microns. **Shade A2**	41,134	

*Bis-GMA *Bisphenol A glycidyl methacrylate, *UDMA *Urethane dimethacrylate, *Bis-MPEPP *2,2-bis(4-methacryloxypolyethoxyphenyl) propane, *TEGDMA *Triethylene glycol dimethacrylate, *S-PRG *Surface pre-reacted glass ionomer

### Sample size calculation

Power analysis ensured a sufficient sample size with color stability as the primary outcome. Hotwani et al. reported ∆E values of 1.211 ± 0.078, 7.386 ± 0.206, and 11.287 ± 0.26734 with various staining solutions [11]. To reject the null hypothesis of equal Group means, 12 specimens per Group were needed. This sample size detects a large effect size (d = 0.86) with 80% power and 5% significance for a two-sided test. The G Power program (version 3.1.9.4) calculated the sample size.

### Ethics consideration

The research was approved by the Research Ethics Committee of the Faculty of Dentistry and the Ethics Committee of Misr University for Science and Technology on 2nd July 2025, with number 2025/0083.

### Study design

Seventy-two disks were divided into three Groups (24 each) by modeling resin application: Group 1 (surface), Group 2 (between layers), and Group 3 (control). Each Group had two subdivisions (12 each): A (color stability) and B (fluoride release). One calibrated operator performed all specimen preparation. Two independent, blinded assessors each evaluated color change or fluoride release to minimize bias. Data collection, verification, and statistical analysis were conducted independently; no preparer or assessor was involved in the analysis.

### Fabrication of Giomer disks

Group 1 (Surface Modeling Resin Application): Giomer was applied per the manufacturer’s instructions into cylindrical split Teflon molds (4 mm height and 10 mm width). The mold was placed over a glass slab and filled incrementally: the first 2 mm layer was cured, then a second 2 mm layer was added. Before curing the second layer, two layers of modeling resin were added in minimal amount to the surface with a fine composite brush simulating the clinical application technique. The procedure was performed to ensure uniform surface coverage and standardization of the manipulation [7]. A celluloid strip and a standardized weight (250 g) were placed over the mold to ensure consistent, void-free specimens, minimize oxygen inhibition, and promote the formation of a smooth, highly polymerized surface. A calibrated light-curing unit (EliparTM Deep Cure-S LED, light output 1200 mW/cm2) was used to light-cure the material for 40 s (double the recommended time), at a 90-degree angle, with the light source positioned 0 mm from the mold to ensure optimal polymerization of the specimen. Doubling the curing time was done as the first increment was 2 mm away from the light-curing tip, with the possibility of light attenuation [6].

Specimens in the other two Groups were prepared similarly but differed in the application of the modeling resin: either between layers or not (control).

Group 2 (In-between Modeling Resin Application): A similar mold was marked at 2 mm. Giomer was placed in the first 2 mm, followed by two layers of modeling resin applied with a composite brush and light-cured. The next 2 mm Giomer was added and light-cured again, as previously described [12, 13].

Group 3 (Control): Giomer was added to the cylindrical mold per the manufacturer’s instructions, then filled and cured in 2 mm increments without modeling resin, as previously described.

**Fig. 1:**
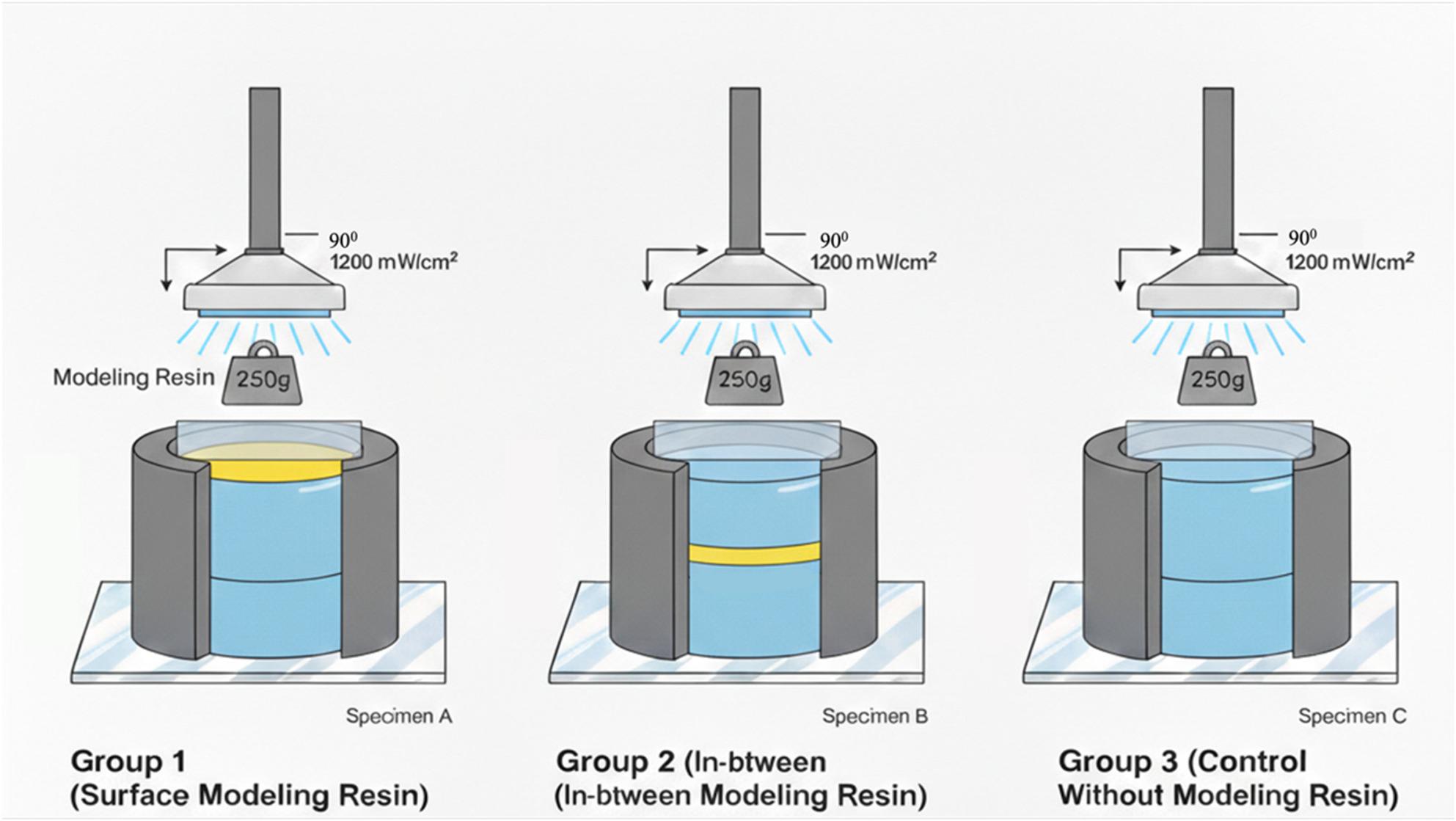
Illuspreparation, two outcome variables were assessed with different evaluation periods: color stability was measured at baseline and after 14 days, while fluoride ion release was assessed over an extended period up to 28 days

The top surfaces of all specimens were finished and polished using a series of aluminum oxide abrasive discs (medium to superfine; Sof-Lex™ Pop-On, 3 M ESPE, USA). To mimic the conditions of the prepared cavity, the disk was coated with two layers of transparent nail varnish on all surfaces except the top. This coating standardized the area exposed to the staining solution [11]. All specimens were coded to avoid assessment bias and stored for 24 h at 37 °C and 100% humidity to facilitate rehydration. Specimen preparation for the different Groups is illustrated in Fig. 1.

Following specimen preparation, two outcome variables were assessed with different evaluation periods: color stability was measured at baseline and after 14 days, while fluoride ion release was assessed over an extended period up to 28 days.

### Color measurement

#### Baseline measurement

After the final setting (24 h of storage in distilled water), specimens were blot-dried, and a recently calibrated Easyshade Advance 4.0 Spectrophotometer (VITA Zahnfabrik, Bad Sackingen, Germany) measured the specimen color according to the Commission Internationale de l’Eclairage values. Measurements were taken from the center of each specimen, against a white background, perpendicular to the specimen surface, at zero distance from the specimen surface, and under D65 lighting conditions to standardize optical conditions and light reflection (ISO 7491 standards for dental material color stability testing). One examiner took all the measurements. Each measurement was repeated three times, and the arithmetic mean was used for analysis [14]. Color variation ΔE* baseline and 14 days of storage was calculated in the 3-dimensional L*a*b* color space, which represented the level of lightness (0 dark, 100 light), the intensity of green/red color, the amount of blue/yellow color, the chroma, and the hue, respectively.

#### Staining protocol

Specimens were subjected to staining challenge after the baseline color measurement. Immersion cycles were as follows: A coffee solution was prepared by combining 20 g of instant coffee (Seelaz, Egypt) with 1000 milliliters of hot water, with a pH of approximately 4.9–5.2. The solution was filtered after stirring for 10 s every 5 min until it cooled to room temperature. Specimens were incubated (CBM.TORRE PICENARDI (CR), Model 431/V, Italy) at 37 °C for 15 min twice a day using a fresh coffee solution each time. After immersion in a coffee solution (25 milliliters of coffee per five specimens), each specimen was ultrasonically cleaned, rinsed, and stored separately in distilled water, which was changed daily, for 14 days [15]. Color was measured under the same conditions as the baseline. Overall color change CIEDE2000 (ΔE 00) was determined for each specimen using the relevant equations.

$$\begin{aligned} \triangle\mathrm E00=&\left[\left(\triangle\mathrm L'/{\mathrm K}_{\mathrm L}{\mathrm S}_{\mathrm L}\right)^2+\left(\triangle\mathrm C'/{\mathrm K}_{\mathrm C}{\mathrm S}_{\mathrm C}\right)^2+\left(\triangle\mathrm H'/{\mathrm K}_{\mathrm H}{\mathrm S}_{\mathrm H}\right)^2 \right. \\& \left. +{\mathrm R}_{\mathrm T}\left(\triangle\mathrm C'/{\mathrm K}_{\mathrm C}{\mathrm S}_{\mathrm C}\right)^2\left(\triangle\mathrm H'/{\mathrm K}_{\mathrm H}{\mathrm S}_{\mathrm H}\right)^2\right]^{1/2} \end{aligned}$$

where ΔL′, ΔC′, and ΔH′ denote the differences in lightness, chroma, and hue between paired specimens, respectively. The weighting functions S_L_, S_C_, and S_H_ were applied to account for variations in visual perception across the color space. The parametric factors K_L_, K_C_, and K_H_ were set to 1 and incorporated into the formula to account for inaccuracies introduced by experimental factors, such as the material’s surface and the background used in the measurement. Everything was performed according to the instructions of ISO/ CIE11664-6:2020. Color changes were additionally interpreted using the 50:50% perceptibility (PT) and acceptability (AT) thresholds, which were set at 0.81 and 1.77, respectively, for CIEDE2000 (1:1:1) calculations [11].

#### Fluoride release assessment by using an Ion Chromatograph (IC)

The other half of the specimens were stored individually in plastic containers with 5 ml of distilled water at 37 °C for 28 days. Solutions were changed daily during the first week. Discs were removed from the solutions, rinsed with double-distilled water, blot-dried with tissue paper, and transferred to new tubes containing 5 mL of fresh solution. At the time of fluoride measurement, each specimen was removed, and the storage solution was collected for analysis. Fluoride release was measured by ion chromatography (IC) at days 1, 3, and 7 during the first week and once a week throughout the other three weeks to determine cumulative concentrations.

Free fluoride ion concentrations were determined using a high-pressure ion chromatography (HPIC) system (Dionex ICS-5000, Thermo Fisher Scientific, Sunnyvale, CA, USA), which was equipped with a suppressed conductivity detector. Chromatographic separation was achieved using a Dionex IonPac™ AS14 analytical column (4 \times 250 mm) preceded by an IonPac™ AG14 guard column. Samples (0.5 mL) were obtained from the storage solutions, and a 250 µL aliquot was then injected into the system via the injection loop. An isocratic flow rate of 1.0 mL/min was maintained. Fluoride ions were identified by integrating peak areas and linearly interpolating against bracketing standards. A calibration curve was constructed from fluoride standard solutions. Each sample was analyzed in triplicate with a detection precision of 0.001 ppm. Final values were normalized to the specimen surface area and expressed as ($\:\mu\:$g/cm2) [16].

### Statistical analysis

Data normality was assessed using the Shapiro–Wilk test. Continuous variables were reported as mean ± standard deviation (SD), along with median, minimum, and maximum values. For comparisons between independent Groups, one-way ANOVA followed by Tukey’s post hoc test was used, while paired t-tests were used for comparisons within related specimens. The threshold for statistical significance was set at *p* < 0.05. All analyses were conducted using IBM SPSS Statistics for Windows, Version 25.0 (IBM Corp., Armonk, NY, 2017).

## Results

### Color parameters

At baseline, L* values were the lowest in Group 1 (Surface Modeling), followed by Group 2 (In Between Modeling), and highest in Group 3 (Control), with a significant difference between Groups (*p* < 0.001); no significant difference was observed between Groups 1 and 2. For a*, the highest mean was in Group 1, followed by Group 3 and Group 2 (*p* = 0.02), with no difference between Groups 2 and 3. b* values were highest in Group 1, followed by Group 3 and the lowest in Group 2 (*p* = 0.047), with no significant difference between Groups 2 and 3 (Table 2).

**Table 2:** Descriptive statistics and the result of ANOVA test and Tukey post hoc test for comparison of color parameters at baseline between the three groups

		Group 1	Group 2	Group 3	*p*-value
L	Mean (SD)	78.9^a^ (0.5)	78.6^a^ (0.6)	82.8^b^ (1.8)	< 0.001*
a	**Mean (SD)**	0.6^a^ (0.2)	0.2^b^ (0.1)	0.4^b^ (0.2)	0.02*
B	**Mean (SD)**	14.4^a^ (0.2)	13^b^ (1.1)	14.1^b^ (1.2)	0.047*

*Significant at *p*≤ 0.05

**Different lower-case letters indicate statistical significance within the same row

At 14 days, L* was the highest in Group 1, followed by Group 3, and the lowest in Group 2 (*p* = 0.001); a* was the lowest in Group 1 compared to Groups 2 and 3 (*p* < 0.001); b* was also the lowest in Group 1 compared to Groups 2 and 3 (*p* < 0.001). In all parameters, no significant differences were noted between Groups 2 and 3 for a* and b* (Table 3).

**Table 3:** Descriptive statistics and the result of ANOVA test and Tukey post hoc test for comparison of color parameters at 14 days between the three groups

		Group 1	Group 2	Group 3	*p*-value
L	Mean (SD)	75^a^ (0.2)	73.9^b^ (0.4)	74.3^b^ (0.5)	0.001*
a	Mean (SD)	0.8^a^ (0.1)	1.3^b^ (0.1)	1.2^b^ (0.2)	< 0.001*
b	Mean (SD)	14.9^a^ (0.3)	15.4^b^ (0.2)	16.4^c^ (0.3)	< 0.001*

*Significant at p≤0.05

**Different lower-case letters indicate statistical significance within the same row

### Changes in color parameters (ΔL, Δa, Δb, ΔE00)

ΔL was the lowest in Groups 1 and 2 and the highest in Group 3 (*p* < 0.001), with no difference between Groups 1 and 2. Δa was highest in Group 2 and Group 3 compared to Group 1 (*p* < 0.001), with no difference between Groups 2 and 3. Δb followed a similar trend: the lowest in Group 1 and the highest in Groups 2 and 3 (*p* = 0.003). ΔE00 was the lowest in Groups 1 and 2 and the highest in Group 3 (*p* < 0.001), with no significant difference between Groups 1 and 2 (Table 4).

**Table 4:** Descriptive statistics and the result of ANOVA test and Tukey post hoc test for comparison of the changes in color parameters between the three groups

		Group 1	Group 2	Group 3	*p*-value
ΔL	Mean (SD)	-3.9^a^ (0.7)	-4.7^a^ (0.6)	-8.6^b^ (2.0)	< 0.001*
Δa	Mean (SD)	0.2^a^ (0.2)	1.0^b^ (0.1)	0.8^b^ (0.3)	< 0.001*
Δb	Mean (SD)	0.5^a^ (0.4)	2.4^b^ (1.1)	2.3^b^ (1.1)	0.003*
ΔE00	Mean (SD)	2.8^a^ (0.5)	3.9^a^ (0.4)	6.3^b^ (1.2)	< 0.001*

*Significant at *p*≤0.05

**Different lower-case letters indicate statistical significance within the same row

### Fluoride release

**Fig. 2:**
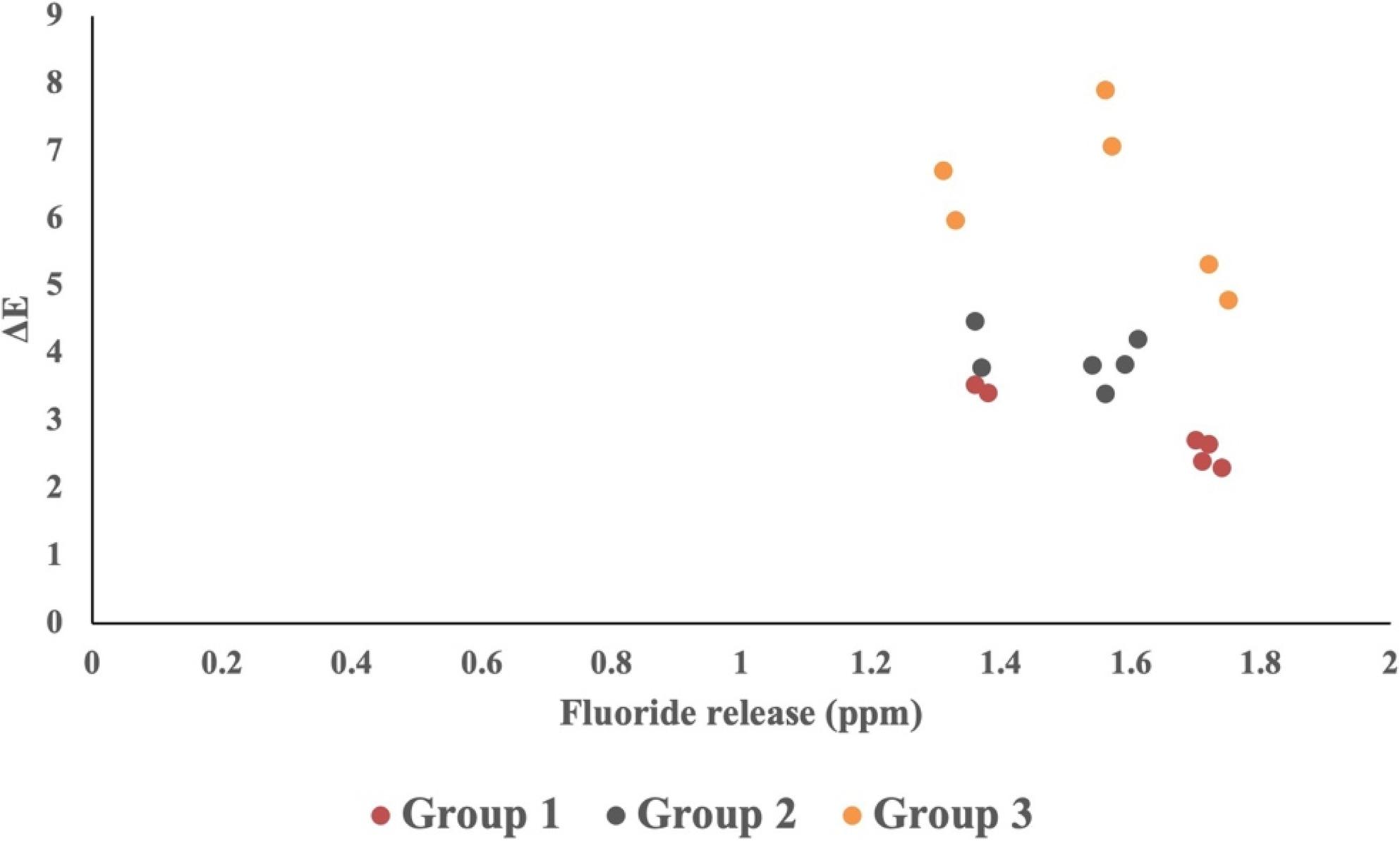
Scatter plot showing the correlation between fluoride release and ΔE

Fluoride release was similar across Groups: Group 1 (1.6 ± 0.2 µg/cm2), Group 2 (1.5 ± 0.1 µg/cm2), and Group 3 (1.5 ± 0.2 µg/cm2) (*p* = 0.593) (Table 5). Pearson’s correlation revealed a weak, non-significant negative correlation between fluoride release and ΔE (*r* = − 0.327, *n* = 15, *p* = 0.185) (Fig. 2).

**Table 5:** Descriptive statistics and the result of one-way ANOVA test for comparison of fluoride release between the three Groups in $\mathrm\mu$g/cm^2^

	Group 1	Group 2	Group 3	*p*-value
*Mean (SD)*	1.6 (0.2)	1.5 (0.1)	1.5 (0.2)	0.593

Non-significant at *p*>0.05

## Discussion

Giomers are hybrid materials that consist of surface pre-reacted glass-ionomer (S-PRG) fillers within the resin matrix. These fillers are produced by an acid–base reaction between fluoroaluminosilicate glass and polyacrylic acid, before incorporation into the resin. The pre-reacted glass-ionomer phase acts as a fluoride reservoir, releasing fluoride ions via water diffusion and ion-exchange mechanisms when exposed to aqueous media. Water sorption into the resin matrix facilitates ion mobility and the gradual diffusion of fluoride from the S-PRG filler surface into the surrounding environment [1, 3]. However, surface treatments and exposure to staining agents may affect their long-term performance [5]. Modeling resin is used to improve the material’s smoothness and reduce its adhesion to the instrument. Whether applied to the surface or between restorative layers, it affects the material’s properties [9, 10]. Evaluating the effects of different application procedures on fluoride release and color stability provides information to optimize the restorative outcomes. Therefore, the current study aims to investigate whether the addition of a modeling resin affects the fundamental functional and cosmetic features of Giomer, providing a basis for clinical recommendations.

In the current study, two application techniques were tested: surface and in-between-layer application to mimic the common clinical techniques and evaluate their effects on the material performance. When applying modeling resin on the top surface of the Giomer, it may affect surface characteristics such as smoothness, surface energy, and porosity, which are critical factors governing stain adsorption and color stability [4]. In contrast, applied resin modeling between restorations may affect the integrity, polymerization, and ion diffusion from the restoration. This comparison provides clinically relevant information to guide practitioners in selecting the most appropriate modeling resin application approach without compromising the material’s bioactive and esthetic properties.

A customized cylinder mold was used in the study. The mold diameter was selected to be 10 mm to exceed the VITA Easyshade measuring tip’s diameter (5 mm), thereby preventing the “edge loss effect,” which occurs when light scatters at the specimen boundaries and compromises brightness measurements [16]. The coffee staining protocol was adapted from the methodology described by Vejendla et al. [15]. Given that the average time to consume one cup of coffee is 15 min, the daily 30-minute immersion cycle employed in this study simulates approximately 1 year of moderate coffee intake [17]. Fluoride release analysis was conducted using Ion Chromatography (IC) rather than the traditional Ion-Selective Electrode (ISE) method to ensure the specific detection of free fluoride ions. Unlike ISE, which measures both free and complexed fluoride, IC facilitates the determination of uncomplexed fluoride species, which are primarily responsible for the cariostatic effect on tooth structure. Additionally, the superior sensitivity of the IC system enabled accurate quantification of trace fluoride concentrations that would otherwise be undetectable via the ISE/TISAB approach [16]. The total cumulative fluoride release was calculated as a single terminal value to compare the materials’ efficacy at the end of the 28 days. This approach was selected to determine the total ion-releasing capacity of the Giomer material when used with modeling resins, focusing on the cumulative therapeutic reservoir rather than short-term release kinetics, as supported by previous methodology [18].

The two different assessment color stability (baseline to 14 days) and fluoride release analysis (28 days) were performed to assess the early change of the Giomer, where both color changes and fluoride release are most pronounced and clinically relevant [18].

The study findings indicated rejection of the null hypothesis regarding color stability, as surface modeling resin improved color stability; however, no association was observed between the application of modeling resin and fluoride release.

Regarding the comparison of baseline color parameters between the three Groups, the result showed significant differences in color parameters (L, a, and b) among the three experimental Groups, which indicates that applying modeling resin influenced the shade of the Giomer (Table 2). The difference may be attributed to the addition of modeling resin, which contains resin, nano-filler, and color pigmentation that may change color [19]. The significantly higher L values in the control Group than in the modeling resin Groups indicate greater lightness when no modeling resin was applied. This finding may be explained by the presence of Bis-GMA resin in the modeling resin’s composition, which may alter light scattering [20]. Regarding a* parameter, Group 1 (Surface Modeling) exhibited significantly higher a* than Groups 2 (In Between Modeling) and Group 3 (Control), indicating a shift toward the red chromatic axis when resin was applied as a surface coating. The result may be attributed to the presence of color pigments on the disk’s surface, which are known to influence wavelength absorption and color saturation [20]. For the b* parameter, the highest values were observed in Group 1 (Surface Modeling), followed by the Control Group, while Group 2 (In Between Modeling) demonstrated the lowest b* values. This variation may be related to differences in yellow chromaticity, caused by the high ceramic filler content and by glass fillers that may alter light-scattering behavior. Also, it may enhance the yellow component due to increased surface resin content [20, 21]. Concerning the values of the color parameters after staining procedures for 14 days, there were statistically significant differences among the three Groups between shades in all studied color parameters (L*, a*, and b*), showing that the mode of application of modeling resin is quite relevant to the optical behavior of restorations during staining (Table 3). After staining, Group 1 (Surface Modeling) had the highest L* value, followed by Group 3 (Control Group), while Group 2 (In Between Modeling) had the lowest L* value. This finding could be due to a surface resin layer that acts as a protective barrier against water sorption and pigment penetration, as well as to ceramic microparticles that improve surface structure [21, 22].

Regarding the a* and b* values after staining procedures, Group 1 (Surface Modeling) demonstrated significantly lower values than Groups 2 (In Between Modeling) and the Control Group, indicating that surface modeling resin application limits oxidative degradation of the resin matrix and reduces stain uptake [23, 24].

The comparison of color parameters at 14 days (L*, a*, b*) agrees with the analysis of color changes (ΔL, Δa, Δb, and ΔE00) (Table 4). Group 1 had the highest L* and lowest a* and b* values after 14 days, resulting in significantly lower Δ values, indicating improved color stability. Groups 2 (In Between Modeling) and 3 (Control Group) had lower L* and higher a* and b* values at 14 days, which corresponded to larger Δ values, indicating stronger darkening and chromatic shifts. At 14 days, there were no significant differences between Groups 2 (In Between Modeling) and the Control Group for a* and b*, which is consistent with the absence of significant differences in Δa and Δb. Similarly, the significantly higher ΔE observed in Group 3 reflects the greater overall color change measured at 14 days, validating the absolute color findings.

These findings were supported by previous studies that confirmed that modeling resin application contributes to the color stability. Researchers have posited that this positive effect may be due to the nano-filler structure and hydrophobic character of the modeling resin. The hydrophobic properties decrease water sorption, preserving color stability [25, 26]. However, this result contradicts Pereira et al., who did not observe a statistical difference in total color change (ΔE) or color parameters (ΔL, Δa, and Δb) after using modeling resin with a composite disk [27]. This discrepancy may be due to differences in filler content: our current study used a modeling resin containing microfilled filler with a ceramic portion of more than 73% micro-fine ceramic, which may provide greater protection for the restoration.

Also, Alebady et al. [28] confirmed that different coat types had no significant influence on fluoride release from glass ionomer restorations, while they contributed to a reduction in surface roughness, which may explain the improvement in color stability [28].

Beyond statistical variations, the clinical performance of color change was assessed using standardized visual thresholds. In esthetic dentistry, the 50:50% perceptibility threshold (PT) represents the point at which an equal number of observers can and cannot detect a color difference. Conversely, the 50:50% acceptability threshold (AT) identifies the limit beyond which the color change is deemed clinically intolerable. In this study, all Groups exceeded the PT (∆E00 > 0.81) after the 14-day staining cycle. While this indicates that the staining was visually detectable, the more critical metric for clinical longevity is whether the values remained below the AT (∆E00 < 1.77). This suggests that while the investigated materials were susceptible to early-stage extrinsic staining, the magnitude of this shift did not reach a level of clinical rejection during the first two weeks of exposure. This finding agreed with that of Paolone et al. [11].

Regarding fluoride release, the results showed no significant difference among the three Groups (Table 5). These findings demonstrated that modeling resin application, whether applied on the surface or between composite increments, did not significantly influence fluoride release. The comparable fluoride release values observed among the three Groups suggest that fluoride ion diffusion is primarily governed by the intrinsic composition of the restorative material rather than any application techniques. Factors such as filler type, fluoride-containing phases, resin matrix chemistry, and ion-leachable glass content appear to play a more decisive role in controlling fluoride release behavior [29].

The absence of a significant effect may also be attributed to the hydrophobic nature of the modeling resin, which could limit water sorption and ion exchange at the Giomer surface [24, 30]. While this hydrophobic barrier may enhance color stability and surface properties, it does not appear to affect fluoride-ion mobility in the evaluated material. These results indicate that modeling resin application can be employed to enhance handling and surface properties without compromising the fluoride ion release of Giomer. The application of modeling resin, consisting of a highly filled micro-fine ceramic structure (> 73% ceramic filler content), reduces the resin matrix in Giomer, providing a more compact structure that limits the diffusion of staining. High filler load also improves wear resistance and reduces resin degradation, resulting in greater color stability without interfering with the ion exchange of Giomer.

A previous systematic review supported the current finding, which concluded that the modeling resin does not hinder fluoride release, confirming that fluoride release depends solely on the filling material type rather than the surface treatment [31]. Also, a 2020 study found that applying coatings to glass ionomer GIC delayed burst release but maintained the fluoride release capacity of GICs at a constant level throughout the tested time periods [32].

The current study has several limitations that must be considered when interpreting the findings. First, it is an in vitro study that may not fully replicate the complex characteristics of the oral environment. Second, only one staining agent (coffee) was used to simulate discoloration, limiting the number of familiar dietary or environmental sources of staining. Third, Vita Easyshade is intended mainly for clinical assessment; the obtained L*, a*, and b* values should be interpreted comparatively rather than as absolute material colorimetric values. Finally, only the amount of fluoride released and color characteristics were measured; other analytical procedures, such as microhardness testing, scanning electron microscopy, or long-term clinical evaluation, might improve our understanding of the long-term impact of modeling resin application.

## Conclusions

Under the conditions of the present study, the following conclusions are gained;

- The integration of modeling resin significantly modifies the baseline chromatic characteristics of Giomer-based restorations, necessitating careful shade selection during clinical application.
- Surface application of modeling resin serves as a protective barrier, significantly enhancing color stability and increasing resistance to extrinsic staining over time.
- The use of modeling resin does not impede the fluoride-releasing capacity of the Giomer material, ensuring that the restorative’s therapeutic benefits remain uncompromised.
- No definitive correlation exists between fluoride release kinetics and color stability.

## Data Availability

The data sets used and/or analyzed in the current research are available upon reasonable request from the corresponding author.

## Abbreviations

UDMA: Urethane Dimethacrylate
Bis-GMA: Bisphenol A Glycidyl Methacrylate
Bis-MPEPP: 2,2-bis(4-methacryloxypolyethoxyphenyl) propane
TEGDMA: Triethylene Glycol Dimethacrylate
S-PRG: Surface Pre-reacted Glass Ionomer
IC: Ion Chromatography
ISE: Ion-Selective Electrode
TISAB: Total Ionic Strength Adjustment Buffer
